# Assessing metarepresentational abilities in adolescence: an exploratory study on relationships between definitional competence and theory of mind

**DOI:** 10.3389/fpsyg.2024.1456432

**Published:** 2024-08-12

**Authors:** Alessia Cornaggia, Federica Bianco, Ilaria Castelli, Carmen Belacchi

**Affiliations:** ^1^Department of Human and Social Sciences, University of Bergamo, Bergamo, Italy; ^2^Department of Communication Sciences, Humanities and International Studies, University of Urbino Carlo Bo, Urbino, Italy

**Keywords:** theory of mind, mindreading, definitional competence scale, adolescence, metarepresentational abilities

## Abstract

**Introduction:**

Several developmental changes occur in adolescence, particularly in the metarepresentational domain, which allows and promotes adaptive sociality. We explored the possible relationships between theory of mind (ToM) and definitional competence, both metarepresentational, beyond age and gender effects.

**Methods:**

To reach our goals, we involved 75 adolescents (age range 14–19 years, *M* = 15.7, and *SD* = 1.36). ToM was measured through “The Reading the Mind in the Eyes Test” (RMET), and definitional competence was assessed through a new instrument, namely, the “Co.De. Scale”. Attention was paid to check whether results were different when considering mental states vs. non-mental states of the scale and emotional words vs. non-emotional words.

**Results:**

*T-tests* showed that older adolescents (third grade of high school) performed better than younger ones (first grade of high school) in both tasks. Only in the male group, there were no school grade differences in the ToM task. Regression analyses showed that RMET performance predicted the score of *non-emotional mental states* definitions and, even if marginally, of *ToM word* definitions. However, RMET was not a predictor of the general performance of the definitional task or *emotion* definitions.

**Discussion:**

Connections with global adolescents’ development and possible educational implications are discussed.

## 1 Introduction

Adolescence is a developmental phase characterized by several changes (Lerner and Steinberg, 2009; Waite-Jones and Rodriguez, 2022), such as major physical transformation, the increase of social relationships, in particular, the relevance of the bond with peers (Carpendale and Lewis, 2004; Zerwas et al., 2004), and also deep cognitive maturation (Byrnes, 2003). In particular, the emotional world in adolescence intertwines with the effects of physical changes that could elicit anxiety, uncertainty, and insecurity; moreover, the experience of these feelings enables self-reflection based on the relational exchanges, especially in the peer group (Palmonari and Crocetti, 2011). This developmental phase is also characterized by complex interactions between interpersonal emotional states, such as guilt, shame, forgiveness, gratitude, self-compassion, and prosocial behavior (Carlo et al., 2023).

Moreover, representational abilities play a crucial role, as identity emerges from the mental representations about oneself and others and also from the meanings attributed to past, present, and possible future experiences (Białecka-Pikul et al., 2020; Bosacki et al., 2020). An important feature of thought in adolescence is the development of metacognitive abilities that consist of “making use of knowledge to remember, reason, make decision and solve problems” (Byrnes, 2003, p. 241), but also the capacity to reflect on one’s own and others’ knowledge (Moshman, 1998). The adolescents’ exercise of thought elaboration allows them to develop personal beliefs, values, and critical abilities that permit them to adapt to their cultural context (Białecka-Pikul et al., 2020).

In this complex framework of changes that involve the acquisition of more abstract levels of representation, in particular, of a metacognitive, metarepresentational, and metalinguistic type, a crucial maturation concerns neural structures and connectivity (Byrnes, 2003; Devine and Lecce, 2021; Laghi and Lonigro, 2022). Although experience could be an input for neural changes, the maturation of frontal lobes (Byrnes, 2003) and the reorganization of the pre-frontal cortex (Blakemore, 2008) seem to specifically contribute to the development of executive functions and metarepresentational abilities, which significantly contribute to the increase of abstract thinking skills during adolescence (Steinberg, 2005).

The type of thought that has as its object the representations of themselves is precisely what characterizes the hypothetical-deductive thought that opens adolescents’ cognitive development to the world of possibilities and inferences (Piaget and Inhelder, 1955). Indeed, what distinguishes Piaget’s formal operational stage is the ability to operate on abstract ideas and knowledge, due to the independence of thought from current action. Although Piaget’s stadial theory does not attribute a primary role to social components in the early stages of development, exposure to others’ perspectives is considered an important element in accessing formal operational thinking (Marchetti and Massaro, 2002). In this process of discovering one’s own and others’ perspectives, Piaget seems to recognize language’s function of triggering reflection about beliefs and mental content. Subsequent evolutions of cognitive psychology have revealed how both adolescents and adults do not follow perfect formal logic in their reasoning, but there are systematic errors that could arise from the mental representations and interpretations of premises in a specific socio-cultural context, as well as from the limits of working memory or linguistic pragmatics (Carugati and Selleri, 2011).

The ability to understand that inferences on mental states allow the prediction and possible explanations of behaviors (Premack and Woodruff, 1978; Wimmer and Perner, 1983), namely ToM is a possible crucial factor intertwined with other metarepresentational abilities that affect the main cognitive and affective domains of adolescents’ experience (Apperly, 2021; Devine and Lecce, 2021).

### 1.1 ToM improvement in adolescence

While traditionally research on ToM has focused primarily on the preschool and school-age periods with the aim of overcoming classical false belief tasks (Wellman, 2012; Castelli et al., 2022), across the past two decades, ToM research has turned into a lifespan perspective (Kuhn, 2000; Marchetti et al., 2016; Peterson and Wellman, 2019), showing that ToM ability continues to undergo relevant changes both on the behavioral and on the neural levels (Castelli et al., 2010; Cabinio et al., 2015). Previous studies also explored associations between ToM and social relations (Sebastian, 2015; Lebedeva et al., 2023), between ToM and the socio-emotional domain (Clifford et al., 2021; Mulvey et al., 2022), and between ToM and both neural (Sebastian et al., 2012; Vetter et al., 2014) and cognitive (Altgassen et al., 2014; Wang et al., 2021) domains during adolescence.

A field of research in the literature on ToM in adolescence is concerned with the validity of measurement tools and measures to assess ToM in adolescence (Hayward and Homer, 2017), such as the Animated Triangle Task (Andersen et al., 2022), the Theory of Mind Assessment Scale (Bosco et al., 2014), EmpaToM—Youth (Breil et al., 2021), and the automated ToM measurement through machine learning and deep learning systems (Devine et al., 2023).

If we consider the most traditional measures of ToM, such as the false belief tasks, Valle et al. (2015) highlighted the development of third-order recursive thinking from adolescence to adulthood. Indeed, in participants aged 14, 17, and 20 years, an age effect on their performances at the third level false belief task was found, controlling for general cognitive abilities. Meanwhile, through the Imposing Memory Task (Kinderman et al., 1998), a set of five stories for advanced ToM that involve the recursive thinking ability within complex social situations and one control story detected a correlation between the third level of reasoning and language comprehension ability. The absence of correlations between lower and upper levels revealed the mastery of first- and second-order reasoning, and the great difficulty with the fourth and fifth levels of reasoning (Valle et al., 2015). The development of recursive thinking in late childhood and adolescence was investigated longitudinally by Van Den Bos et al. (2016) on participants aged from 8 to 17 years. The authors observed both cross-sectionally and longitudinally (after 2 years) an effect of age on the recursive thinking ability that seemed to follow a linear development up to the age of 18 years. Moreover, verbal reasoning could play a role in the development of recursive thinking more than vocabulary (Van Den Bos et al., 2016). These results were in line with a previous study by Dumontheil et al. (2010) in a sample of females aged from 7 to 27 years, where researchers highlighted that the perspective-taking ability continued its improvement even in late adolescence when the development of executive functions had already reached the levels of adulthood (Dumontheil et al., 2010). Based on the measures of this study, Symeonidou et al. (2016) used eye-tracking in a sample composed of children, adolescents, and adults, detecting a difference in the adults’ ToM performances when compared with younger participants, a result that seems to confirm adolescence as an ongoing developmental phase in reasoning about own and others mental states.

Beyond these results, which mainly concern the cognitive aspects of ToM, the research on ToM development in adolescence also focused on the affective components of mental state reasoning and its social use (Bosacki, 2015). In particular, the longitudinal results by Bialecka-Pikul et al. (2020) in a sample of 13- and 16-year-old Polish adolescents showed an increase in psychological self-descriptions from early to middle adolescence and also a between-group significant difference in advanced ToM. A measure frequently used to assess affective ToM in its socio-perceptual component in the lifespan is the RMET (Baron-Cohen et al., 1997, 2001). Such measures showed significant results in studies with adolescents (Meinhardt-Injac et al., 2020; Gabriel et al., 2021). Meinhardt-Injac et al. (2020) detected a specific effect of age on ToM, which was not related to improvements in other cognitive abilities, such as language and executive functions, in participants aged between 11 and 25 years. More specifically, Gabriel et al. (2021) focused on the development of both affective and cognitive ToM in three phases of adolescence: early (13–14 years), middle (15–16 years), and late (17–18 years) adolescence. The results showed lower performance in both ToM components by early adolescents, whereas no significant changes emerged when comparing between middle and late adolescents. Moreover, in the first period of adolescence, a relationship between affective ToM and verbal abilities, such as fluency, flexibility, and verbal intelligence, was observed, while cognitive ToM was found to be related to language comprehension in all the considered age groups (Gabriel et al., 2021). This study also highlighted better performances in cognitive ToM in female participants compared with male participants. Gender differences in ToM in adolescence are still controversial: evidence in favor of female students has been found in advanced ToM measures (Bosco et al., 2014; Białecka-Pikul et al., 2017, 2020; Gabriel et al., 2021), but they have not always been confirmed (Bosacki et al., 2020). Moreover, possible influences of gender stereotypes, such as a higher social awareness of girls than boys, have to be considered (Bosco et al., 2014; Białecka-Pikul et al., 2021). The study conducted by Białecka-Pikul et al. (2021) also detected an association between advanced ToM and verbal abilities that was particularly evident in female participants. Bosco et al. (2014), using the Theory of Mind Assessment Scale (Th.o.m.a.s.—Bosco et al., 2009), highlighted better performances by female participants in a sample of pre-adolescents and adolescents from 11 to 17 years. In line with the previous illustrated studies, the authors showed an effect of age on ToM development that became more stable in participants older than 15 years (Bosco et al., 2014). Moreover, the longitudinal study conducted by Stępień-Nycz et al. (2021) explored changes in advanced ToM in 13- and 16-year-old participants, observing a female better performance in advanced ToM measured through the Ambiguous Story Task (Bosacki et al., 2015), controlling for language.

As mentioned in previous studies, another important variable that interacts with ToM development is language in its different components and skills (Antonietti et al., 2006; Siegal and Surian, 2011; Pinto et al., 2017; de Villiers, 2021). The studies on first- and second-order ToM development have highlighted a reciprocal relationship between these two areas in the course of development (Belacchi, 2022; Miller, 2022), but in adolescence, the picture is far from clear. The lower number of studies in advanced or mature ToM combined with the increasing complexity of ToM abilities in middle childhood and adolescence does not help to clarify the relationship between ToM and language in adolescents (Milligan et al., 2007; Devine and Lecce, 2021), even if some results have suggested the possible persistence, even at more advanced levels of development, of some kind of relationship between these two abilities (Antonietti et al., 2006; Im-Bolter et al., 2016; Gabriel et al., 2021). In the complex relationship between language and ToM, another relevant variable to consider is mental-state language, which includes those terms that refer to the cognitive, emotional, and volitional spheres (Lecce, 2009). Studies on middle-childhood ToM development detected a significant association between engagement in mental-state conversations with peers or teachers and ToM abilities in students (Ornaghi et al., 2014; Bianco and Lecce, 2016; Bianco et al., 2019, 2021; Lombardi et al., 2022; Bianco and Castelli, 2023). Indeed, mental-state language allows both the observation of the first expressions of ToM and the understanding of the interactional development of different metarepresentational abilities (Meins et al., 2006; Lecce and Pagnin, 2007). Im-Bolter et al. (2016), in a study that involved participants aged between 7 and 12 years old, detected two different models of interactions between higher-order ToM and other cognitive domains, respectively, for middle childhood and early adolescence. Regarding this second life period, the authors highlighted a less complex model characterized in particular by a lower involvement of mental attentional capacity measured in both its verbal and visuospatial components. This is probably due to the greater experience with ToM reasoning and the development of higher cognitive skills. Moreover, they found a decreased relevance of syntactic language abilities (a closed system), in favor of a persistent role played by the semantic component (an open system), considered a language competence with a higher developmental potential in adolescence and adulthood. Some studies also investigated the possible connections between language and ToM, considering even the social components; for instance, Brodsky et al. (2023) detected a role of language in adolescents’ performance in interpreting unambiguous social scenarios. Lavoie and Talwar (2022) highlighted the role of ToM in determining the tendency of adolescents to maintain transparency and sharing of information with both parents and friends. Widening the perspective, Pluck et al. (2021) investigated the role of adolescents’ SES on language ability, related to ToM and executive function, showing a stronger association with language than with ToM or executive function. The association between ToM and executive function also became non-significant when language was added as a control variable.

In line with these suggestions, language and ToM could be considered important metarepresentational functions that deserve to be further investigated in adolescence, as they evolve in the interaction with the set of complex changes and acquisitions that characterize this phase of life.

### 1.2 Definitional competence in adolescence

Language plays a key role in adolescence: expressing one’s own point of view, discussing, arguing, and exercising critical thinking are crucial skills (Tolchinsky and Berman, 2023). Therefore, metalinguistic and metarepresentational functions, specifically expressed through definitional activity, become fundamental, especially because they allow using language to reflect on language itself in order to share, in a decontextualized way, the semantic representations with anyone who knows our language (Belacchi, 2022). In particular, producing a lexicographic definition, such as a sentence that expresses the meaning (*definiens*) of a given word (*definiendum*), requires combining content and form components (Benelli et al., 2006), but above all, a de-contextualizational perspective that takes into account the interpersonal culturally shared meaning of words (Benelli et al., 2006; Belacchi and Benelli, 2017, 2021). Conventionally, a lexicographic definition, in order to pursue the necessary communicative effectiveness, must assume the Aristotelian format: “An X is a Y that Z,” where “X” represents the given object or concept, “Y” represents the *genus proximum* (the superordinate category), and Z represents the *differentia specifica*, i.e., information that allows the specific object or concept to be identified (Benelli et al., 2006). In particular, a lexicographic definition must have five requisites or rules: a correct and syntactically autonomous linguistic structure (*correctness and morpho-syntactic autonomy rule*), a semantic correspondence between the stimulus item and the sentence that explains its meaning (*semantic equivalence rule*), expressed by verbs such as, “means” “refers to,” “is” (*copula rule*), an articulated sentence (*periphrasis* or *phrasal extension rule*) and the use of different words from the *definiendum* (*no-tautology rule*) (Benelli et al., 2006; Belacchi and Benelli, 2017, 2021). In order to reach such a high level of complex skills, both of form and content, as those required in a definitional task, a process of development in a bidirectional influence with formal instruction is required (Benelli et al., 2006; Artuso et al., 2021). The developmental trend of definitional competence and its specific components can be assessed through the Competence Definitional Scale, Co.De. Scale, (Benelli et al., 2006; Belacchi and Benelli, 2017), whose last version (Belacchi and Benelli, 2021) is structured in seven progressive levels that reflect different degrees of definitional ability. Refer to Table 1 for more details on the levels of the Co.De. Scale. The recent normative study of the development of definitional competence in the Italian population through the Co.De. Scale (Belacchi and Benelli, 2021) showed a significant age improvement (from preschoolers to adults) without significant gender differences. Of note, Dourou et al. (2020), assessing the ability to define words from preschoolers to adults, found a vantage in the female participants in all age groups.

**Table 1:** Definitional levels, prototypical answers, and scores for the definition of the word “Donkey.”

Levels	Kinds of answers	Score
0. Non-definition	Non-verbal answers	0
I. Pre-definition	One-word answers, mostly associations (e.g., *donkey- > ears*)	1
II. Quasi-definition	The initial formulation of sentences, without autonomous forms (e.g., *donkey- > with the long ears; when it brays*)	2
III. Narrative/descriptive definition	Formally correct and autonomous sentences, with narrative/descriptive content (e.g., *donkey brays; donkey is mild*)	3
IV. Categorical definition	Formally correct and autonomous sentences in simply categorical/ synonymic form (e.g., *The donkey is an animal*)	4
V. Partial aristotelian definition	Formal correctness without semantic equivalence (e.g., *The moon is a planet in the solar system*)	5
VI. Aristotelian, metalinguistic definition	Formal and semantic correctness and equivalence (e.g., *A donkey is an animal that brays*)	6

It has been well-documented that definitional competence undergoes significant changes across the lifespan and education levels. The studies by Belacchi and Benelli (2007, 2017, 2021), starting from the first proposals by Litowitz (1977), Watson (1985, 1995), Johnson and Anglin (1995), and Nippold (1995), have detected a development in the quality of definitions from the preschool age to adulthood. Around the age of 7 years, children start to systematically use superordinate categorical terms (“ISA structure”) and not only the so-called “HAS structure” descriptive definitions that characterize the preschool years (Benelli et al., 2006). Around the age of 10–12 years, the quality of definitions becomes similar to the one at adult levels (Benelli et al., 2006). Interestingly, the 11-year-old participants performed better than adults with low educational levels, suggesting the possible role of daily practice with definitions and school learning in determining the quality of definitions (Snow et al., 1989; Snow, 1990). A relevant contribution to the understanding of the developmental process of definitional competence was provided by Belacchi and Benelli (2017) with a study that involved participants aged from 5 to 20 years, divided into five age groups. The results showed not only an increase in lexical knowledge, but also a growing appropriateness, completeness, and formal correctness in comparing all the considered age groups. Recently, a study conducted on the elderly population showed a decline in definitional competence associated with aging (Bianco et al., 2022) and also in the taxonomic organization of representations, a fundamental ability for definitional competence (Belacchi and Artuso, 2018). The study by Bianco et al. (2022) also showed how ToM and definitional competence are related to aging. In particular, ToM was detected as a predictor of definitional competence; moreover, that work showed that definitional competence seems to decline earlier than ToM. In this study, ToM was measured using the Reading the Mind in the Eyes Test (Baron-Cohen et al., 1997, 2001) and the definitional competence through the Co.De. Scale (Belacchi and Benelli, 2021).

Important implications of metarepresentational skills have also been observed in relational and social development, particularly in dealing with the complex phenomenon of bullying (Belacchi and Benelli, 2020). In participants aged between 8 and 10 years, lower levels of definitional competence, particularly related to emotion terms, were associated with hostile roles and predicted aggressive behavior (Belacchi and Benelli, 2020). Moreover, in this study, a positive correlation between two different metarepresentational skills (definitional competence and the cognitive component of empathy) was observed. The common meta-representational nature of cognitive, affective, and relational skills, as well as their influence on social dynamics, are issues that may provide interesting insight into development in adolescence, when relationships, especially with peers, are fundamental for wellbeing (Bagwell and Schmidt, 2013).

In this view, and to the best of our knowledge, the present study focused on definitional competence—a peculiar expression of metalinguistic and metarepresentational skills—in adolescence and on its possible connections with ToM.

As suggested by this theoretical framework and previous studies in the literature, the development of metarepresentational skills (i.e., ToM and definitional competence) could play a crucial role in the significant changes that occur in cognitive, emotional, and social domains during adolescence. Indeed, the ability to understand, reflect, and share mental representations about the connections between internal states and reality was associated with self-understanding processes (Białecka-Pikul et al., 2020), such as the quality of friendship, relationships (Fink, 2021), and social behavior (Bosacki, 2021) in adolescence. Moreover, definitional competence has some connections with social outcomes in children aged between 8 and 10 years (Belacchi and Benelli, 2020), which encourages exploring its connection with other developmental domains in adolescence.

### 1.3 Research questions and hypothesis

The present study aims to deepen our understanding of the development of metarepresentational abilities in adolescence, in particular of a component of the ToM construct, i.e., the ability to infer mental states from eye images, and the definitional competence (regarding both ToM and non-ToM words) as an expression of metalinguistic and metarepresentational abilities.

The first specific aim is to investigate possible differences in the development of ToM and of definitional competence between early and middle adolescence, due to the absence of studies in literature focused on this development phase, to the best of our knowledge. The plurality of changes that occur in adolescence, as shown in previous literature, could be more stabilized in middle adolescence: therefore, we expect better performances from the older group on both tasks measuring the two abilities.

Second, following the evidence concerning gender differences in development during adolescence, we explore possible gender specificities. Despite the absence of corroborating evidence in the existing literature, some suggestions support the hypothesis that female subjects may exhibit superior performance compared to their male counterparts (Bosco et al., 2014; Longobardi et al., 2016; Gabriel et al., 2021).

Finally, we investigate the relationship between the two metarepresentational abilities involved in the study, and, in line with Bianco et al. (2022), we hypothesize that ToM could be a predictor of definitional competence, especially in defining ToM words.

## 2 Materials and methods

### 2.1 Participants and research context

The research involved 75 adolescents (33 males and 42 females) aged from 14 to 19 years (*M* = 15.7 *SD* = 1.36). The research was conducted in the center of Italy, involving three different public high schools: lyceum (*N* = 31), the technical institute (*N* = 19), and the professional institute (*N* = 25). Inclusion criteria were fluency in the Italian language, the absence of neuro-developmental or psychiatric disorders, and attendance in the first and third grades of high school. A total of 36 participants attended the first grade and 39 attended the third grade. The classes were selected on the proposal of school leaders, who assessed the willingness of teaching staff to collaboratively engage. The informed written parental consent and participant’s consent were obtained. Data were collected in the first part of the school year. All requirements of ethical guidelines provided by the Declaration of Helsinki (World Medical Association, 2013), the American Psychological Association (APA, 2017), and the Italian Psychological Association (AIP, 2022) were amended.

### 2.2 Measures

#### 2.2.1 Theory of mind

The Reading the Mind in the Eyes Test (RMET) (Baron-Cohen et al., 1997, 2001) in its Italian version (Serafin and Surian, 2004) was chosen to assess the ability to infer complex emotional and epistemic mental states from images of the eye area of human faces. A total of 18 stimuli (*α* = 0.341)^1^ were presented to participants and subjects were required to select which mental state was represented in the eyes image. For each image, four options, in the form of a single word, were displayed. One point was given for each correct answer. For instance, when looking at an eyes picture, participants have to choose the correct answer: *giocoso* (playful) excluding the wrong labels *confortante* (comforting) *irritato* (irritated) *annoiato* (bored). The total score ranged from 0 to 18.

#### 2.2.2 Definitional competence

The Co.De. Scale (Belacchi and Benelli, 2021) is used to assess seven progressive levels in the ability to define 32 different target words (*α* = 0.884). Participants were required to provide the meaning of eight nouns (caring, spying, rivalry, donkey, clown, orange, skill, and umbrella), eight adjectives (innocent, thin, round, risky, polite, blond, smooth, and contagious), eight verbs (to think, to tolerate, to force, to frustrate, to beat, to burn, to join, and to emigrate), equally distributed between abstract and concrete terms, and eight terms referring to emotions (pride, sadness, anger, shame, envy, guilt, joy, and fear). Each written answer is attributed scores from 0 (non-definition level) to 6 (Aristotelian, metalinguistic definition) along the following scale (an example of response and related score is illustrated in Table 1). After assigning a score (ranging from 0 to 6), to each answer, we calculated the sum of different scores: a general measure or A “*Total*” score that comprised all items, and some more specific scores that were created by distinguishing “*Non-ToM words*” (donkey, to beat, to burn, clown, thin, round, risky, to join, orange, polite, to emigrate, blond, skill, smooth, umbrella, and contagious) from “*ToM words*” (pride, sadness, anger, shame, envy, guilt, joy, fear, caring, innocent, to think, spying, to tolerate, to force, rivalry, and to frustrate), as made in a previous study in the same domain (Bianco et al., 2022). In order to better understand the connections between definitional competence and the multicomponential nature of ToM, the other two scores were calculated, by separating words describing “*Emotions*” (pride, sadness, anger, shame, envy, guilt, joy, and fear) from words referring to “*Non-emotional mental states*” (caring, innocent, to think, spying, to tolerate, to force, rivalry, and to frustrate). In doing that, we have followed the mental-state language categorization proposed by Lecce and Pagnin (2007).

### 2.3 Procedure

Data collection was performed at school. Tasks were collectively administered in classrooms under the presence of the researcher in one session lasting approximately 1 h. To guarantee that each individual was able to work autonomously, the desks were positioned at a distance from one another. Answers were provided in written format. Participants were reassured about the absence of any form of evaluation. The RMET was the first task presented, followed by the definitional competence task.

### 2.4 Analysis

The collected data have been analyzed using Jamovi version 1.6.23 statistical software (The Jamovi Project, 2022). Independent-sample t-tests were used to examine the possible effects of school grade and gender on both definitional competence and ToM. Pearson’s correlations and partial correlations (inserting age as a control variable) were used to explore possible relations between the ToM component measured by RMET and each of the Co.De. Scale scores. To better understand the possible associations between ToM and definitional competence, hierarchical regressions were performed, entering at Step 1 age and, at Step 2, the hypothesized predictor.

## 3 Results

Table 2 presents the descriptive statistics of the considered measures. After we consider age and gender differences in definitional competence and ToM tasks, the last section analyzes the relationships between the two abilities.

**Table 2:** Descriptive statistics of RMET and the Co.De. Scale measures (total, non-ToM words, ToM words, emotions, and non-emotional mental states).

	RMET	Co.De. total	Non-ToM words	ToM words	Emotions	Non- emotional mental states
*M*	11.3	117	59.2	58.1	31.6	28.9
*SD*	2.14	20.2	8.26	13	8.35	7.86
*Min*	7	75	42	33	13	7
*Max*	15	160	76	84	45	44
*Sk*	−0.04	0.01	0.05	0.08	−0.478	−0.44
*Ku*	−0.35	−0.30	−0.54	−0.49	−0.832	0.34

### 3.1 Definitional competence and ToM: age and gender differences

As shown in Table 3, all scores showed better performances by the third-grade students compared with the first-grade students. However, results seemed to suggest an association between age and gender because, considering separately male and female groups (Table 4), in the female group, third-grade students performed better than first-grade students in both metarepresentational measures included in the present study (*p* = 0.019, for RMET and *p* = 0.001, for the Co.De. Scale). In the male group, the only significant difference between first- and third-grade students was the better performance of older male participants in *non-emotional mental states* score, *p* = 0.041.

**Table 3:** Independent sample *t*-test on RMET and the Co.De. Scale scores measured in two school grades.

	Grade 1 (*N* = 36)		Grade 3 (*N* = 39)		Student *t*	*df*
	*M*	*SD*	*M*	*SD*		
RMET	10.81	2.55	11.95	2.55	−2.13*	73
Co.De. total	112.81	20.57	129.23	20.03	−3.50***	73
Non-ToM words	58.53	7.85	62.87	8.96	−2.23*	73
ToM words	54.28	14.11	66.36	12.39	−3.95***	73
Emotions	28.64	8.54	34.36	7.25	−3.14**	73
Non-emotional mental states	25.64	7.40	32	7.06	−3.81***	73

Welch t, equal variances not assumed. *p < 0.05, **p < 0.01, ***p < 0.001.

**Table 4:** Independent sample *t*-test on RMET and the Co.De. Scale scores in two gender groups.

	Grade 1 (*N* = 36)			Grade 3 (*N* = 39)			Student *t*	*df*
	*N*	*M*	*SD*	*N*	*M*	*SD*		
RMET								
Girls	16	10.3	2.41	26	11.5	2.82	−2.44 *	40
Boys	20	11.2	1.64	13	12.2	2.43	- 0.30	31
Co.De. total								
Girls	16	110.88	22.43	26	132.96	18.69	−3.45***	40
Boys	20	114.35	19.41	13	121.77	21.27	−1.03	31
Non-ToM words								
Girls	16	58.13	7.51	26	64.42	8.92	−2.35*	40
Boys	20	58.85	8.29	13	59.77	8.53	−0.308	31
ToM words								
Girls	16	52.75	16.60	26	68.54	11.20	−3.69***	40
Boys	20	55.50	12.07	13	62	13.92	−1.42	31
Emotions								
Girls	16	27.2	9.47	26	36.2	5.88	−3.79***	40
Boys	20	29.8	7.77	13	30.8	8.56	−0.337	31
Non-emotional mental states								
Girls	16	25.6	8.52	26	32.4	6.57	−2.92**	40
Boys	20	25.7	6.61	13	31.2	8.20	−2.14*	31

Welch t, equal variances not assumed. *p < 0.05, **p < 0.01, ***p < 0.001.

### 3.2 Relation between definitional competence (the Co.De. Scale) and the ability to read mental state from gaze (RMET)

Table 5 illustrates the correlations between RMET and all the scores on the Co.De. Scale. As we can observe, the expected associations between the ToM score and different definition scores, even if all positive, were not significant. Only correlations between ToM and some specific Co.De. scores were statistically significant. In particular, between RMET and *ToM Words* in the Co.De. Scale (*r =* 0.271, *p* = 0.019), and RMET and *Non-emotional mental states* (*r* = 0.277, *p* = 0.016). This last positive correlation remains significant, *r =* 0.234, *p* = 0.045, also adding age as a control variable.

**Table 5:** Bivariate and partial correlations (weighted for age) among RMET and the Co.De. Scale measures.

	RMET	Co.De. total	Non-ToM words	ToM words	Emotions	Non-emotional mental states
RMET	1	0.194	0.035	0.271*	0.208	0.277*
Co.De. total	0.148	1	0.900***	0.965***	0.854***	0.872***
Non-ToM words	−0.003	0.897***	1	0.755***	0.641***	0.710***
ToM words	0.227^+^	0.962***	0.744***	1	0.900***	0.886***
Emotions	0.170	0.844***	0.623***	0.894***	1	0.596***
Non-emotional mental states	0.234*	0.860***	0.696***	0.874***	0.565***	1

In the lower part of the matrix, we have the partial correlations (weighted for age). *^+^* < 0.1, *p < 0.05, **p < 0.01, ***p < 0.001.

In order to better understand the relationship between the two metarepresentational abilities, we performed a series of regression analyses. Table 6 shows the regression analysis on the Co.De. Scale scores. We entered age at Step 1, adding RMET at Step 2. Regarding the *Total* score of the Co.De. Scale, Step 1 was significant, *F*(1, 73) = 6.51, *p* = 0.013, while Step 2 did not increase the variance explained, *ΔF*(1, 72) = 1.62, and *p* = 0.21. When we investigated the impact of ToM on definitional competence, separating *non-ToM* and *ToM words*, the pattern of results was different. In *ToM words* score, as seen for the *total* score, Step 1 (i.e., age) was significant, predicting *ToM words*, *F*(1, 73) = 7.78, *p* = 0.007, β = 3.32, *t* = 2.79, and *p* = 0.007. Step 2 led to a marginally significant increase in the variance explained, *ΔF*(1, 72) = 3.90, *p* = 0.052. Differently, age and RMET were not predictors of *non-ToM words*. In order to deepen the significant results on *ToM words*, it is interesting to observe the pattern of results of the sub-scores. For what concerns, *non-emotional ToM words* both Step 1, *F*(1, 73) = 7.93, and *p* = 0.006, and Step 2, *F*(2, 72) = 6.22, and *p* = 0.003, were significant, with an increase in the significant variance explained by RMET, Δ*F*(1, 72) = 4.17, and *p* = 0.045. On the other hand, for *emotions*, only Step 1 was significant [*F*(1, 73) = 4.61, *p* = 0.035], and Step 2 did not increase the variance explained, Δ*F*(1, 72) = 2.14, *p* = 0.15.

**Table 6:** Hierarchical regressions predicting definitional competence.

		*R*^2^	*B*	*SE*	*β*	*t*	*p*
Co.De. total							
Step 1	Age	0.08	4.60	1.80	0.29	2.55	0.013
Step 2	Age	0.10	4.16	1.83	0.26	2.27	0.026
	RMET		1.33	1.04	0.15	1.27	0.207
Non-ToM words							
Step 1	Age	0.04	1.28	0.73	0.20	1.75	0.085
Step 2	Age	0.04	1.28	0.75	0.20	1.71	0.092
	RMET		−0.01	0.43	−0.00	−0.03	0.979
ToM words							
Step 1	Age	0.10	3.32	1.19	0.31	2.79	0.007
Step 2	Age	0.14	2.87	1.19	0.27	2.42	0.018
	RMET		1.34	0.68	0.22	1.98	0.052
Emotions							
Step 1	Age	0.06	1.50	0.70	0.24	2.15	0.035
Step 2	Age	0.09	1.31	0.71	0.21	1.85	0.069
	RMET		0.59	0.40	0.17	1.46	0.148
Non-emotional mental states							
Step 1	Age	0.10	1.82	0.65	0.31	2.82	0.006
Step 2	Age	0.15	1.57	0.64	0.27	2.44	0.017
	RMET		0.75	0.37	0.23	2.04	0.045

We also investigated the opposite direction, i.e., the Co.De. Scale scores as predictors of RMET. Specifically in Step 1, we entered age, and in Step 2, the scores of the Co.De. Scale in each regression analysis were entered. In this case, Step 1 was never significant, *p_s_* ≥ 0.103. Interestingly, *ToM words* Co.De. Scale (marginally), *β* = 0.23, *t* = 1.98, and *p* = 0.052, and *non-emotional ToM words* Co.De. Scale*, β* = 0.24, *t* = 2.04, and *p* = 0.045, both predicted the RMET score, with the following increases in variance*: ΔR*^2^ = 0.05. Other relations were all non-significant.

To sum up, age was a predictor of *Total* Co.De. Scale score and sub-scores: *ToM words, non-emotional ToM words,* and *emotions*. RMET was a predictor of *non-emotional ToM words* and there was a marginally significant result for the *ToM words* sub-score of the Co.De. Scale. Considering the opposite direction, *ToM words* and *non-emotional ToM words* sub-scores of the Co.De. Scale were, respectively, marginally significant and significant predictors of RMET.

## 4 Discussion

This study, though preliminary, aims to explore the relations between ToM and definitional competence in adolescence, integrating the developmental processes of metarepresentational abilities into the broader complexity of changes that lead adolescents to build their personal and social identities. There are no previous studies specifically investigating definitional competence in adolescence through the Co.De. Scale or its relationship with ToM in this period of life.

In line with what was hypothesized, the results showed better performances of older groups of participants both in RMET and in all scores of the Co.De. Scale. The significance of age as a predictor for multiple Co.De. Scale scores could depend on multiple factors. One of these could be the role played by formal education in the improvement of students’ reasoning and language skills (Benelli et al., 2006; Dourou et al., 2020; Artuso et al., 2021). In particular, third-grade students could be already more familiar with the different metarepresentational abilities involved in high school grade education, which for first-grade students could be an ongoing challenge to deal with (Byrnes, 2003). Another factor could be represented by brain structural maturation, with increases in connectivity in the prefrontal, temporal, and temporoparietal areas that characterize the adolescence phase (Devine and Lecce, 2021; Gabriel et al., 2021; Laghi and Lonigro, 2022). Moreover, as suggested by other evidence that detected differences between early and middle adolescence, the higher tendency of early adolescents to prioritize self-perception over the effort to understand others (Bosco et al., 2014) could have affected, in the younger group, both the ability to infer mental states from the eyes of another person and the accuracy in providing clear information in defining terms. Our findings about ToM are in line with those of Gabriel et al. (2021), who observed a significant increase in both cognitive and affective ToM between 13- and 16-year-olds, suggesting a higher cognitive demand for younger participants, especially in terms of attention. In addition, Bialecka-Pikul et al. (2020), exploring the interactions between advanced ToM and self-construction, highlighted the relation between ToM and the process of self-construction, finding significant improvement in both developmental dimensions in adolescents from 13 to 16 years. Of note, we observed a particularly high value of SD for the Co.De. Scale. This indicates the presence of high variability of performance in our sample for definitional competence. Even if this result is preliminary and should be confirmed in future research, a possible explanation could be related to the relevant changes in cognitive skills that happen during adolescence (Carugati and Selleri, 2011).

The second specific aim of this study was to explore possible gender differences in our sample, given that previous results were rather mixed in their conclusions on this issue (Bosco et al., 2014; Białecka-Pikul et al., 2017; Dourou et al., 2020; Belacchi and Benelli, 2021; Stępień-Nycz et al., 2021; Caputi and Bosacki, 2023). Our results partially confirm the better performances of female participants compared with male participants because, in the ToM task, we observed the above-mentioned age group’s significant difference only in the female group. In agreement with what was hypothesized in other studies (Bosco et al., 2014; Gabriel et al., 2021), this difference could be explained by a different development of neuropsychological processes and structures, but also by socio-cultural factors and competences. Even in a study conducted by Longobardi et al. (2016) on mental state language in primary schools, a better performance of female participants in the use of terms related to mental states was detected, although male participants obtained higher scores than female participants in receptive language. In order to better understand these gender differences that are not consistent in the literature, further studies are needed that could confirm these findings and more fully explore the possible variables involved.

The third and more innovative purpose of this study focuses on the possible associations between the two metarepresentational abilities: ToM and definitional competence. Consistent with a similar study conducted by Bianco et al. (2022) in aging, RMET performances also predicted the score of *ToM words* definitions in adolescence. However, in the present study, RMET was neither a predictor of general performance in the definitional task nor on *emotion* definitions. The only definitional scores that have been significantly predicted by the RMET score were the *non-emotional mental states* score and, even if marginally, the *ToM words* score. Reciprocally, none of the definitional scores was a predictor of RMET performance. These results confirm the presence of some subtle, but not negligible, associations between the implicit metarepresentational ability to infer mental states and to produce lexicographic definitions also in adolescence, stressing the link between ToM and language competence, often remarked in literature (de Villiers, 2021). In particular, Gabriel et al. (2021) highlighted the role of verbal ability in facilitating the process of affective ToM reasoning knowing the meaning of emotion words. Similarly, Im-Bolter et al. (2016) highlighted in adolescence the possible interaction between ToM and semantic components of language. As suggested in the previous studies with older people (Bianco et al., 2022), the specific association between RMET and the Co.De. Scale could be in the direction of moving from the implicit nature of the ToM task to the explicit nature of metarepresentational processes necessary to produce a high-level definition. The unexpected lack of significant associations between RMET and the definitions of emotions could be explained by the emotional turbulence that characterizes the adolescent phase, which can make conscious access to emotions, and the consequent conventional verbalization, which is particularly difficult (Marchetti and Cavalli, 2013; Gatta et al., 2014; Muzi, 2020).

This study presents some limitations related to the need to segment the complexity of developmental processes while reading the results obtained within a systemic framework (Bronfenbrenner, 1979) that takes into account the mutual interactions between the multiple domains and sources of development. The first limitation of the study is ToM measurement, which, especially for advanced forms of mental state reasoning, would require the employment of a battery composed of multiple and more articulated tools that would allow for consideration of different components of the ToM construct (Beaudoin et al., 2020), such as the cognitive and affective ToM (Gabriel et al., 2021). Similarly, language should be examined not only in its metalinguistic aspect as a definitional competence but also through the employment of multiple instruments that investigate different linguistic competences. The third important limitation is the sample size; thus, future studies will need the recruitment of a larger sample to derive more accurate conclusions. Finally, a limitation regards the lack of information about participant social and affective life or their specific abilities in other cognitive and emotive domains. Future studies could broaden the perspective in these directions, with the aim of placing the development of metarepresentational abilities within a more comprehensive framework that investigates reciprocal associations of different domains. It could be useful in the future to apply a longitudinal approach to the issues investigated here.

## 5 Conclusion

In summary, this study deals with the complexity of development in adolescents through the investigation of implicit and explicit levels of metarepresentational abilities. The major innovative element was the exploration of the use of the Co.De. Scale in this specific age period, which added new knowledge to the relationship between metalinguistic skills and ToM. Definitional competence could already represent a construct that allows for an increase in the understanding of the interaction between language and the ability to infer mental states. For this reason, it could be interesting in future studies to include multiple measurements of ToM to explore the possible different connections of definitional competence and different components of ToM. Because of the relevance of social and relational dimensions in adolescence, it could be worth deepening the role of these two metarepresentational abilities in interaction with other key factors of development, such as the quality of social interactions, emotion recognition, and social adjustment. For instance, it may be beneficial to consider ways to support the development of metarepresentational skills as a potential way to assist adolescents in navigating the emotional challenges that accompany the many changes they experience during this period of life. Increasing knowledge about specific metarepresentational abilities and the relationship between them could also set the basis for the implementation of training studies with multiple possible educational implications, as suggested also by Belacchi and Benelli (2020) for what concerns the connections of definitional competence and empathy with bullying roles in primary school children. Adolescence could be a sensitive period for prevention and people’s wellbeing, but programs should be based on a detailed understanding of individual differences and should include the promotion of multiple skills (Caputi and Bosacki, 2023). For example, as observed by studies concerning the “Promoting mental health at schools” (PROMEHS) program (Cefai et al., 2022; Colomeischi et al., 2022; Martinsone et al., 2022), processes of social and emotional learning could play a role for adolescents’ wellbeing/prosocial behavior, and these competences are also likely to increase resilience in critical situations such as those that occurred during the pandemic. Moreover, the linguistic and metalinguistic abilities that enable an individual to represent and socially share their internal states could be relevant factors, especially for the development of social and emotional learning (Cavioni et al., 2017). We hope our study can help in exploring these directions.

## Data Availability

The raw data supporting the conclusions of this article will be made available by the authors, without undue reservation.

## Footnotes

^1^The low index for RMET, a widely used measure in ToM domain, is in line with previous studies (Olderbak et al., 2015; Hayward and Homer, 2017).
